# Examining the association of habitual e-cigarette use with inflammation and endothelial dysfunction in young adults: The VAPORS-Endothelial function study

**DOI:** 10.18332/tid/162327

**Published:** 2023-06-12

**Authors:** Ellen Boakye, S. M. Iftekhar Uddin, Ngozi Osuji, Jill Meinert, Olufunmilayo H. Obisesan, Mohammadhassan Mirbolouk, Erfan Tasdighi, Omar El-Shahawy, John Erhabor, Albert D. Osei, Tanuja Rajan, Michael Patatanian, Janet T. Holbrook, Aruni Bhatnagar, Shyam S. Biswal, Michael J. Blaha

**Affiliations:** 1 Johns Hopkins Ciccarone Center for Prevention of Cardiovascular Disease, The Johns Hopkins University, Baltimore, United States; 2 The American Heart Association Tobacco Regulation and Addiction Center, University of Louisville, Dallas, United States; 3 Department of Medicine, Brookdale University Hospital Medical Center, New York City, United States; 4 Department of Internal Medicine, University of Pittsburg Medical Center, Pittsburg, United States; 5 Department of Epidemiology, Johns Hopkins Bloomberg School of Public Health, Baltimore, United States; 6 Department of Medicine, MedStar Union Memorial Hospital, Baltimore, United States; 7 Department of Population Health, New York University Grossman School of Medicine, New York, United States; 8 Department of Biostatistics, Johns Hopkins Bloomberg School of Public Health, Baltimore, United States; 9 Department of Medicine, University of Louisville School of Medicine, Louisville, United States; 10 Department of Environmental Health and Engineering, Johns Hopkins Bloomberg School of Public Health, Baltimore, United States

**Keywords:** e-cigarette, endothelial dysfunction, inflammation, flow-mediated dilation, reactive hyperemia index

## Abstract

**INTRODUCTION:**

Acute exposure to e-cigarette aerosol has been shown to have potentially deleterious effects on the cardiovascular system. However, the cardiovascular effects of habitual e-cigarette use have not been fully elucidated. Therefore, we aimed to assess the association of habitual e-cigarette use with endothelial dysfunction and inflammation – subclinical markers known to be associated with increased cardiovascular risk.

**METHODS:**

In this cross-sectional study, we analyzed data from 46 participants (23 exclusive e-cigarette users; 23 non-users) enrolled in the VAPORS-Endothelial function study. E-cigarette users had used e-cigarettes for ≥6 consecutive months. Non-users had used e-cigarettes <5 times and had a negative urine cotinine test (<30 ng/mL). Flow-mediated dilation (FMD) and reactive hyperemia index (RHI) were used to assess endothelial dysfunction, and we assayed high-sensitivity C-reactive protein, interleukin-6, fibrinogen, p-selectin, and myeloperoxidase as serum measures of inflammation. We used multivariable linear regression to assess the association of e-cigarette use with the markers of endothelial dysfunction and inflammation.

**RESULTS:**

Of the 46 participants with mean age of 24.3 ± 4.0 years, the majority were males (78%), non-Hispanic (89%), and White (59%). Among non-users, 6 had cotinine levels <10 ng/mL while 17 had levels 10–30 ng/mL. Conversely, among e-cigarette users, the majority (14 of 23) had cotinine ≥500 ng/mL. At baseline, the systolic blood pressure was higher among e-cigarette users than non-users (p=0.011). The mean FMD was slightly lower among e-cigarette users (6.32%) compared to non-users (6.53%). However, in the adjusted analysis, current e-cigarette users did not differ significantly from non-users in their mean FMD (Coefficient=2.05; 95% CI: -2.52–6.63) or RHI (Coefficient= -0.20; 95% CI: -0.88–0.49). Similarly, the levels of inflammatory markers were generally low and did not differ between e-cigarette users and non-users.

**CONCLUSIONS:**

Our findings suggest that e-cigarette use may not be significantly associated with endothelial dysfunction and systemic inflammation in relatively young and healthy individuals. Longer term studies with larger sample sizes are needed to validate these findings.

## INTRODUCTION

Over the past decade, there has been a tremendous increase in the use of e-cigarettes in the United States, particularly among adolescents and young adults^1,2^. E-cigarettes are now the most popular tobacco product among youth in the US, surpassing combustible cigarette use in 2014 ^3,4^. In 2022, approximately 2.55 million US middle and high school students reported past-30-day e-cigarette use ^5^. Among young adults aged 18–20 years and 21–24 years, the prevalence of past-30-day e-cigarette use in 2020 was 15.6% and 14.5%, respectively ^6^.

Although studies have shown that e-cigarette aerosol, compared to combustible cigarette smoke, has lower levels of toxicants, the health effects of e-cigarettes, particularly after long-term use, are not fully known ^7,8^. While some studies have shown the potential acute cardiovascular toxicity of e-cigarettes, their long-term cardiovascular effects have not been fully described ^9-12^. Acute exposure to e-cigarette aerosol has been associated with a shift in cardiac autonomic balance toward sympathetic predominance, increased oxidative stress, and reduced flow-mediated dilation (FMD) ^12,13^, which are all changes that are known to be strongly associated with increased cardiovascular risk ^14,15^.

FMD, defined as vasodilation in response to an increase in luminal blood flow and reactive hyperemia index (RHI), are validated measures for assessing endothelial dysfunction ^16,17^. Impaired FMD and RHI are early changes in subclinical vascular injury and have both been associated with increased incidence of cardiovascular events ^18,19^. Similarly, inflammation, which can be induced by nicotine, is an early measure of vascular disease that has been shown to be associated with increased cardiovascular risk ^20-22^.

To fill knowledge gaps in the cardiovascular effects of habitual e-cigarette use, we conducted a pilot study assessing the association of habitual e-cigarette use with markers of endothelial dysfunction and inflammation. Addressing this gap is crucial in informing the debate on the benefits versus risks of e-cigarettes as well as their regulation.

## METHODS

### Study design and participants

The VAPing Observational Research Study-Endothelial function (VAPORS-E) is a sub-study of the VAPORS study designed to study the long-term effects of e-cigarette use on oral microbiome and oral health. Individuals aged 18–34 years who were in good health and were not current combustible cigarette smokers were eligible for the VAPORS study. Participants were recruited from the Johns Hopkins University campuses, other Maryland college campuses, vape shops, and surrounding areas using flyers and social media advertisements. Eligible and recruited participants in the parent VAPORS study also qualified for the VAPORS-E study. VAPORS-E was a single-visit study, and eligible participants consented and tested on the same day. The protocols for the Oral VAPORS study and the VAPORS-E study were reviewed and approved by the Johns Hopkins Institutional Review Board.

### Exposure assessment

E-cigarette users were required to have a minimum of 6 consecutive months of e-cigarette use. Non-users had used e-cigarettes <5 times cumulatively, had not used an e-cigarette in the past 90 days and had a negative urine cotinine test (<30 ng/mL) ^23^. In addition, both e-cigarette users and non-users were not current combustible cigarette smokers or current users of other tobacco products. Additional inclusion and exclusion criteria for the parent study are provided in Supplementary file Table 1. Participants had their urinary cotinine levels assayed and categorized as: 0–10, 10–30, 30–100, 100–200, 200–500, 500–1000, and >1000 ng/dL. A total of 49 participants aged 18–34 years were enrolled in VAPORS-E, consisting of 23 exclusive e-cigarette users and 26 age-and sex-matched non-users. Among the non-users, 3 participants were excluded due to high cotinine levels (>30 ng/dL).

### Outcome assessment

Endothelial function was assessed in the Johns Hopkins Cardiovascular Clinical Research Unit using brachial FMD and RHI. FMD was assessed non-invasively using high-resolution brachial artery ultrasound probe (Toshiba Aplio; Ultrasound transducer PLT-1202S 12MHz). Brachial FMD (imaged 2 cm above antecubital fossa; forearm occlusion; cuff pressure to 250 mmHg; occlusion for 5 mins) was assessed by the same experienced technician for all participants. Change in the brachial artery diameter post-occlusion was measured, and the absolute per cent change from baseline was calculated. The Johns Hopkins Clinical Research Unit reports reproducibility statistics of FMD assessment of 98–99% (intra-observer variability for repeated measurements is 2 ± 1%) ^24^. RHI was measured with the EndoPAT (Itamar Medical, Israel), a noninvasive technique that uses blood pressure cuff occlusion and fingertip probes to measure changes in the digital pulse volume ^25^.

Serum measures of inflammation in this study included high-sensitivity C-reactive protein (hs-CRP), interleukin-6 (IL6), fibrinogen, p-selectin, and myeloperoxidase. The biomarkers were assayed at the Johns Hopkins Clinical Research Core Laboratory. Details of the assays used in measuring the inflammatory markers and measures of variability are provided in Supplementary file Table 2. The serum samples used to assess the biomarker levels were taken at the same time of exposure assessment (e-cigarette use assessment and cotinine measurement). All study participants fasted for at least 10 hours and had not taken caffeine or alcohol 24 hours prior to FMD and RHI assessment. The technicians who assessed the FMD and RHI at our clinical research unit, as well as inflammatory markers at the central research laboratory, were blinded to the participants’ e-cigarette use status.

### Covariate assessment

Participants’ sociodemographic characteristics including age, sex, race (White, Black/African American, Asian, American Indian/Alaskan Native), ethnicity (Hispanic, non-Hispanic), marital status (married, single, living with significant other, single, not living with significant other), employment (employed for wages, self-employed, out of work, student), and education level (8th Grade or less, more than 8th Grade but not a high school graduate, high school graduate or equivalent), were assessed at enrollment in this study.

### Statistical analysis

The study participants were first stratified into e-cigarette users and non-users. Then, their characteristics were summarized using proportions for categorical variables, means with standard deviation for normally distributed continuous variables, and medians with interquartile range (IQR) for non-normally distributed continuous variables. Next, differences in proportions were tested using the chi-squared and Fischer’s exact tests. In contrast, differences in normally distributed and skewed continuous variables were tested using Student’s t-test and Mann-Whitney rank sum test. Skewness and kurtosis tests were used to check the normality assumption on the distributions of our outcome measures.

We assessed the association of e-cigarette use with endothelial dysfunction and inflammation using linear regression models. FMD and RHI values in our study followed a normal distribution. Models were first unadjusted (Model 1), then age-adjusted (Model 2), and finally adjusted for age, sex, race, education level, ever-combustible cigarette use, body mass index (BMI), systolic blood pressure (SBP), and diastolic blood pressure (DBP) (Model 3). The selection of confounders was based on literature and biological plausibility. E-cigarette users were further categorized by their cotinine levels (≤1000, and >1000 ng/dL). In all analyses, non-users were used as the reference group.

In assessing the association between e-cigarette use and inflammatory markers, we first evaluated each marker separately (log-transformed due to skewness of the distribution) and subsequently as part of a cumulative inflammatory marker score. For each inflammatory marker, a participant was assigned a score of 1 when levels were ≥75th percentile for the study sample. Since five inflammatory markers were assessed, scores ranged 0–5. We further categorized this score into 0/1 vs ≥2 elevated markers. Using Poisson regression with robust variance, we evaluated the association of e-cigarette use with having ≥2 elevated inflammatory markers.

All analyses were conducted using Stata version 16 (StataCorp, College Station, TX). A 2-sided alpha (α) level of <0.01 (0.05/5 for inflammatory marker analysis) and 0.025 (0.05/2 for endothelial function analysis) were used to determine statistical significance.

## RESULTS

Of the 46 participants (mean age: 24.3 ± 4.0 years), the majority (78.3%) were males, non-Hispanic (89.1%), and White (58.7%). Compared to non-users, those who used e-cigarettes were younger (23.0 vs 25.6 years; p=0.027) but had a higher mean BMI (26.9 vs 23.9 kg/m^2^; p=0.021) and higher mean SBP (116.9 vs 107.8 mmHg; p=0.011). While only 1 of 23 non-users (4.4%) had ever used combustible cigarettes, 13 of 23 e-cigarette users (28.3%) reported lifetime use (Table 1). The e-cigarette use patterns among e-cigarette users are presented in Supplementary file Table 3. The median duration of e-cigarette use among e-cigarette users was 24 months (IQR: 9–48). Of the 23 non-users, 6 had cotinine levels <10 ng/dL, while the remaining 17 had levels 10–30 ng/dL. Most of the e-cigarette users (14 of 23) had cotinine levels ≥500 ng/dL (Table 1).

**Table 1:** Characteristics of study participants in the cross-sectional VAPORS-Endothelial function study among young adults, 2017–2019

*Characteristics*	*Total (N=46) n (%)*	*Non-users (N=23) n (%)*	*Current e-cigarette users (N=23) n (%)*	*p*
**Age** (years), mean ± SD	24.3 ± 4.0	25.6 ± 4.0	23.0 ± 3.7	0.027
**Male sex**	36 (78.3)	18 (78.3)	18 (78.3)	1.00
**Ethnicity**				0.35
Hispanic	5 (10.9)	4 (17.4)	1 (4.4)	
Non-Hispanic	41 (89.1)	19 (82.6)	22 (95.6)	
**Race**				0.19
White	27 (58.7)	10 (43.5)	17 (73.9)	
Black/African American	5 (10.9)	3 (13.0)	2 (8.7)	
Asian	5 (10.9)	4 (17.4)	1 (4.4)	
American Indian/Alaskan Native	9 (19.5)	6 (26.1)	3 (13.0)	
**Employment**				0.27
Employed for wages	20 (44.4)	8 (34.8)	12 (54.6)	
Self-employed	1 (2.2)	1 (4.3)	0 (0.0)	
Out of work	1 (2.2)	0 (0.0)	1 (4.6)	
Student	23 (51.1)	14 (60.9)	9 (40.9)	
**Marital status**				0.025
Married	4 (8.7)	2 (8.7)	2 (8.7)	
Single, living with significant other	9 (19.6)	1 (4.3)	8 (34.8)	
Single, not living with significant other	33 (71.7)	20 (87.0)	13 (56.5)	
**Education level**				<0.001
Eighth Grade or lower	5 (10.9)	1 (4.4)	4 (17.4)	
More than 8th Grade but not a high school graduate	20 (43.5)	4 (17.4)	16 (69.6)	
High school graduate or equivalent	21 (45.6)	18 (78.3)	3 (13.0)	
**Health status**				
BMI (kg/m^2^), mean ± SD	25.4 ± 4.5	23.9 ± 3.5	26.9 ± 4.9	0.021
Ever smoker	13 (28.3)	1 (4.4)	12 (52.2)	0.001
Baseline heart rate (bpm), mean ± SD	65.4 ± 1.7	62.6 ± 1.8	68.2 ± 2.8	0.11
Systolic blood pressure (mmHg), mean ± SD	112.6 ± 11.8	107.8 ± 12.1	116.9 ± 9.9	0.011
Diastolic blood pressure (mmHg), mean ± SD	66.6 ± 9.4	64.9 ± 9.2	68.2 ± 9.5	0.26
Brachial artery diameter (mm), mean ± SD				
Basal (diastolic)	3.99 ± 0.61	3.94 ± 0.53	4.04 ± 0.69	0.585
Post-occlusion (diastolic)	4.23 ± 0.62	4.19 ± 0.53	4.27 ± 0.70	0.675
Flow mediated dilation (%), mean ± SD	6.42 ± 4.02	6.53 ± 3.98	6.32 ± 4.15	0.87
Reactive hyperemia index, mean ± SD	2.23 ± 0.65	2.17 ± 0.51	2.28 ± 0.16	0.58
hs-C-reactive protein (μg/mL), median (IQR)	0.92 (0.55–1.53)	0.84 (0.55–1.53)	1.09 (0.33–1.75)	0.61
Interluekin-6 (pg/mL), median (IQR)	2.01 (1.33–2.90)	1.71 (1.14–2.11)	2.41 (1.52–3.31)	0.011
Fibrinogen (μg/mL), median (IQR)	3135 (2965–3706)	3344 (3063–3738)	3090 (2797–3534)	0.24
P-selectin (ng/mL), median (IQR)	56.38 (38.17–85.60)	55.43 (37.65–74.77)	57.95 (40.10–96.94)	0.31
Myeloperoxidase (ng/mL), median (IQR)	2.40 (1.83–3.17)	2.36 (1.83–3.17)	2.36 (1.83–3.17)	0.80
**Number of inflammatory markers** ≥**75th percentile**				0.76
0 or 1	29 (63.0)	15 (65.2)	14 (60.9)	
≥2	17 (37.0)	8 (34.8)	9 (39.1)	
**Cotinine levels** (ng/mL)				
0–10		6 (26.1)	-	
10–30		17 (73.9)	2 (8.7)	
30–500		-	7 (30.4)	
500–1000		-	4 (17.4)	
>1000		-	10 (43.5)	

**Figure 1:**
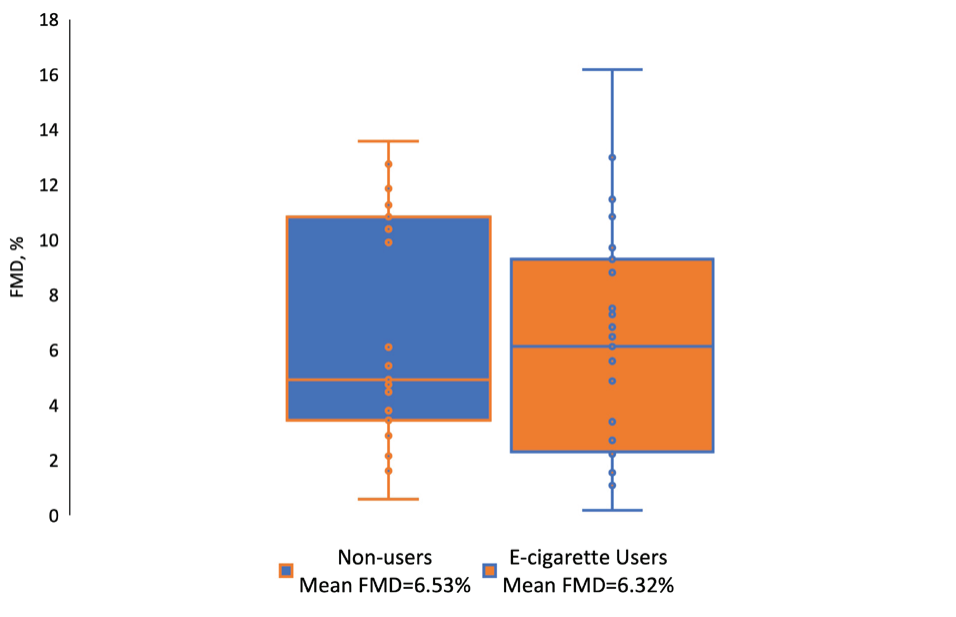
Distribution of flow-mediated dilation by e-cigarette use status

The mean FMD was numerically lower among e-cigarette users (6.32%) compared to non-users (6.53%), however, this difference was not statistically significant (p=0.87) (Figure 1). The mean RHI was 2.28 among e-cigarette users compared to 2.17 among non-users (p=0.58). Compared to non-users, e-cigarette users had higher levels of interleukin-6 (median: 2.41 vs 1.71 pg/mL; p=0.011) and slightly higher, although non-significant, levels of hs-CRP (median: 1.09 vs 0.84 μg/mL; p=0.61) and p-selectin (median: 57.95 vs 55.43 ng/mL; p=0.31). Approximately 39.1% of individuals who vaped had ≥2 inflammatory markers with levels ≥75th percentile, compared to 34.8% of non-users, albeit not significantly different (Table 1).

**Figure 2:**
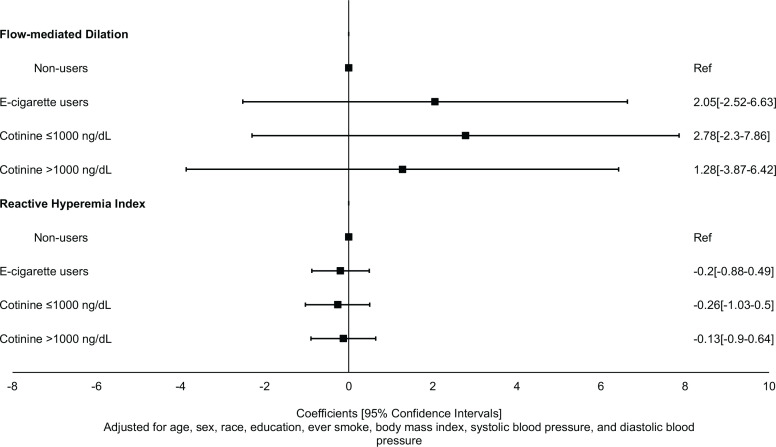
Association of e-cigarette use with measures of endothelial dysfunction

In analysis adjusting for age, sex, race, education level, ever-combustible cigarette use, BMI, SBP, and DBP, current e-cigarette users did not differ significantly from non-users in their mean FMD (Coefficient=2.05; 95% CI: -2.52–6.63) and RHI (Coefficient: -0.20; 95% CI: -0.88–0.49) (Figure 2; Table 2). Similarly, after adjusting for age, sex, race, education level, ever-combustible cigarette use, BMI, SBP, and DBP, current e-cigarette users did not differ significantly from non-users in their mean hs-CRP (Coefficient= -0.68; 95% CI: -1.63– 0.26), IL6 (Coefficient= -0.04; 95% CI: -0.51–0.43), fibrinogen (Coefficient= -0.16; 95% CI: -0.38–0.07), p-selectin (Coefficient=0.05; 95% CI: -0.45–0.56), and myeloperoxidase (Coefficient=0.15; 95% CI: -0.79–1.09) (Table 3).

**Table 2:** Association of e-cigarette use with measures of endothelial dysfunction

*Measure*	*Model 1*		*Model 2*		*Model 3*	
	*Coefficient (95% CI)*	*p*	*Coefficient (95% CI)*	*p*	*Coefficient (95% CI)*	*p*
**FMD**						
Non-users (n=23) (Ref.)						
Current e-cig users (n=23)	-0.20 (-2.62–2.12)	0.87	-0.40 (-2.99–2.18)	0.75	2.05 (-2.52–6.63)	0.37
Cotinine ≤1000 ng/dL (n=13)	0.19 (-2.68–3.06)	0.90	-0.04 (-3.14–3.07)	0.98	2.78 (-2.30–7.86)	0.27
Cotinine >1000 ng/dL (n=10)	-0.71 (-3.8–2.43)	0.65	-0.81 (-4.03–2.40)	0.61	1.28 (-3.87–6.42)	0.62
**RHI**						
Non-users (n=20) (Ref.)						
Current e-cig users (n=22)	0.11 (-0.30–0.53)	0.58	-0.06 (-0.47–0.35)	0.78	-0.20 (-0.88–0.49)	0.56
Cotinine ≤1000 ng/dL (n=13)	0.08 (-0.40–0.56)	0.75	-0.15 (-0.62–0.33)	0.54	-0.26 (-1.03–0.50)	0.49
Cotinine >1000 ng/dL (n=9)	0.17 (-0.37–0.71)	0.54	0.05 (-0.46–0.56)	0.84	-0.13 (-0.90–0.64)	0.73

Model 1: crude. Model 2: adjusted for age. Model 3: adjusted for age, sex, race, education level, ever smoker, BMI, systolic blood pressure, and diastolic blood pressure.

**Table 3:** Association of e-cigarette use with inflammatory markers

*Marker*	*Model 1*		*Model 2*		*Model 3*	
	*Coefficient (95% CI)*	*p*	*Coefficient (95% CI)*	*p*	*Coefficient (95% CI)*	*p*
**hsCRP**						
Non-users (Ref.)						
Current e-cig users	0.18 (-0.45–0.82)	0.57	0.32 (-0.35–0.98)	0.35	-0.68 (-1.63–0.26)	0.15
Cotinine ≤1000 ng/dL	0.07 (-0.69–0.82)	0.86	0.23 (-0.57–1.04)	0.57	-0.66 (-1.71–0.39)	0.21
Cotinine >1000 ng/dL	0.33 (-0.49–1.16)	0.42	0.41 (-0.42–1.24)	0.32	-0.71 (-1.78–0.36)	0.18
**IL6**						
Non-users (Ref.)						
Current e-cig users	0.39 (0.11– 0.68)	0.008	0.42 (0.11–0.72)	0.008	-0.04 (-0.51–0.43)	0.86
Cotinine ≤1000 ng/dL	0.40 (0.05–0.74)	0.024	0.43 (0.06–0.80)	0.024	-0.02 (-0.54–0.51)	0.95
Cotinine >1000 ng/dL	0.39 (0.02–0.76)	0.039	0.41 (0.03–0.79)	0.036	-0.06 (-0.59–0.46)	0.80
**Fibrinogen**						
Non-users (Ref.)						
Current e-cig users	-0.04 (-0.18–0.09)	0.50	-0.05 (-0.19–0.09)	0.46	-0.16 (-0.38–0.07)	0.16
Cotinine ≤1000 ng/dL	-0.02 (-0.18–0.14)	0.79	-0.03 (-0.20–0.14)	0.74	-0.11 (-0.36–0.13)	0.36
Cotinine >1000 ng/dL	-0.08 (-0.25–0.10)	0.38	-0.08 (-0.26–0.10)	0.37	-0.20 (-0.45–0.05)	0.11
**P-selectin**						
Non-users (Ref.)						
Current e-cig users	0.13 (-0.16–0.43)	0.37	0.23 (-0.07–0.53)	0.13	0.05 (-0.45–0.56)	0.83
Cotinine ≤1000 ng/dL	-0.01 (-0.35–0.34)	0.97	0.11 (-0.25–0.46)	0.56	-0.04 (-0.60–0.52)	0.88
Cotinine >1000 ng/dL	0.31 (-0.06–0.69)	0.10	0.37 (0.00–0.74)	0.05	0.15 (-0.42–0.72)	0.59
**Myeloperoxidase**						
Non-users (Ref.)						
Current e-cig users	-0.16 (-0.71–0.38)	0.55	-0.20 (-0.79–0.38)	0.49	0.15 (-0.79–1.09)	0.75
Cotinine ≤1000 ng/dL	-0.32 (-0.97–0.32)	0.32	-0.39 (-1.09–0.31)	0.27	-0.12 (-1.14–0.90)	0.81
Cotinine >1000 ng/dL	0.04 (-0.67–0.74)	0.91	0.01 (-0.72–0.73)	0.99	0.44 (-0.60–1.47)	0.40

All the inflammatory markers are log-transformed. Model 1: crude. Model 2: adjusted for age. Model 3: adjusted for age, sex, race, education level, ever smoker, BMI, systolic blood pressure, and diastolic blood pressure.

The adjusted prevalence of having ≥2 inflammatory markers with levels ≥75th percentile did not differ significantly between current e-cigarette users and non-users (adjusted prevalence ratio, APR=1.11; 95% CI: 0.17–7.06) (Table 4). In all analyses, stratifying current e-cigarette users by their cotinine levels did not change the inference of our findings.

**Table 4:** Association of e-cigarette use with inflammatory marker score

*≥2 Inflammatory markers ≥75 th percentile*	*APR (95% CI)*	*p*
Non-users (Ref.)		
Current e-cig users	1.11 (0.17–7.06)	0.92
Cotinine ≤1000 ng/dL	0.62 (0.07–5.36)	0.67
Cotinine >1000 ng/dL	1.90 (0.25–14.29)	0.53

APR: adjusted prevalence ratio; adjusted for age, sex, race, education level, ever smoker, BMI, systolic blood pressure, and diastolic blood pressure.

## DISCUSSION

In this small sub-study of 46 healthy young adults, habitual exclusive e-cigarette use was not associated with marked changes in markers of endothelial dysfunction or inflammation, although e-cigarette users had higher systolic blood pressure at baseline. Therefore, our findings suggest that e-cigarette use in healthy young adults may not be significantly associated with endothelial dysfunction and systemic inflammation, although small effect sizes cannot be excluded, given our small sample size.

Acute exposure to e-cigarette aerosol, even from non-nicotine devices, has been shown to be associated with increases in oxidative stress, endothelial dysfunction, and inflammation ^26^. Several preclinical and some clinical studies have shown that exposure to e-cigarette aerosol could be a potential cardiovascular health concern as it causes decreased intravascular and released nitric oxide and increased oxidative stress, both mechanisms that lead to vascular endothelial dysfunction ^9,10,13,27,28^. In a study by Mohammadi et al.^28^ among 120 healthy volunteers with a mean age of approximately 30 years, sera from chronic e-cigarette users (>5 times/week for >3 months) had reduced vascular endothelial growth factor (VEGF)-induced nitric oxide secretion compared to sera from non-users. Contrary to the findings of our study, the aforementioned study found that chronic e-cigarette users had significantly lower mean FMD compared to non-users and a mean FMD comparable to combustible cigarette smokers ^28^.

The observed differences in our study outcomes compared to the study by Mohammadi et al.^28^ may stem from the relatively younger participants used in our study. In our study, the mean ages of e-cigarette users and non-users were 23.0 ± 3.7 years and 25.6 ± 4.0 years, respectively, compared to 29.0 ± 4.6 years and 28.0 ± 4.0 years in the study by Mohammadi et al.^28^. Additionally, the stricter selection of non-users in the study by Mohammadi et al. ^28^, i.e. <1 pack-year of smoking and >5 years of smoking cessation if ever smoked and never daily marijuana smoking, may have resulted in a relatively healthier control than the control subjects used in our study. However, similar to the findings of our study, Majid et al.^29^ found no differences in baseline FMD between regular e-cigarette (JUUL) users and non-users, although acute e-cigarette use significantly decreased FMD compared to non-use. Also, in a study by Fetterman et al.^30^, baseline FMD did not differ between chronic e-cigarette users and non-users. Thus, while several preclinical studies have shown that exposure to the use of e-cigarettes causes endothelial dysfunction, evidence among clinical subjects in the non-acute use setting has generally been contradictory. It is important to add that other novel tobacco products, such as heat-not-burn cigarettes, have been found to be associated with impaired endothelial function, similar to findings among combustible cigarette smokers.^31^

E-cigarette use has also been linked to systemic inflammation with increased inflammatory biomarkers such as IL-6, IL-8, and matrix metalloproteinase-9 in e-cigarette users compared to non-users ^32^. In our study, e-cigarette users had moderately higher levels of IL-6 than non-users. Similarly, other studies have found very little to no significant differences in the levels of inflammatory biomarkers among e-cigarette users compared to non-users ^33,34^. In a large study among 7130 participants of the Population Assessment of Tobacco and Health (PATH) Study, there was no difference in the biomarker concentration of inflammatory (high-sensitivity C-reactive protein, interleukin-6, fibrinogen, soluble intercellular adhesion molecule) and oxidative stress (urinary 8-isoprostane) between participants who used e-cigarettes and non-users ^34^.

Thus, while acute exposures to e-cigarettes may impact the cardiovascular system with a shift in cardiac autonomic balance toward sympathetic predominance and oxidative stress ^9-12^, the findings of our study suggest that chronic exposures, particularly in healthier young adults, may not significantly be associated with large changes in endothelial function and systemic inflammation. Alternatively, any potential long-term cardiovascular risk associated with e-cigarette use may be mediated by mechanisms other than endothelial dysfunction and systemic inflammation. Such mechanisms may include hemodynamic changes, heart rate variability, increased susceptibility to arrhythmias, and increased platelet aggregation and adhesion ^35,36^.

### Strengths and limitations

A strength of our study is that the FMD and RHI were assessed by the same technician in an experienced academic lab among all study participants, thereby reducing variability. Additionally, e-cigarette use or non-use status was verified by urinary cotinine levels. However, the limitations of our study include its non-randomized observational nature, which limits causal inferences and the potential for residual confounding. Also, our small study sample consisted of very healthy young adults with favorable socioeconomic characteristics; hence our findings may not be generalizable to the general population. Finally, the inherent variability in FMD measurements may slightly hamper its use as a biomarker of endothelial function. However, impaired FMD is generally accepted as a measure of endothelial dysfunction and has been shown to be significantly associated with cardiovascular risk ^37^.

## CONCLUSIONS

Taken together, the findings of our study suggest that among healthy young adults, e-cigarette use may not be significantly associated with endothelial dysfunction and systemic inflammation, as assessed by the markers used in this study. However, well-powered longitudinal studies with longer follow-up and detailed assessments of e-cigarette use patterns are needed to make more conclusive observations about the cardiovascular health impacts of chronic e-cigarette use. Finally, future meta-analyses of all studies that have assessed chronic e-cigarette use with markers of endothelial dysfunction and systemic inflammation are strongly encouraged. This would provide a more precise estimate of the effect size and increase the generalizability of results.

## Data Availability

The data supporting this research are available from the authors on reasonable request.
