# Supplementary material for: Examining the association of habitual e-cigarette use with inflammation and endothelial dysfunction in young adults: The VAPORS-Endothelial function study
Source: Tob Induc Dis. 2023 Jun 12;21:75. doi: 10.18332/tid/162327 (PMC10257221; doi:10.18332/tid/162327)
Supplement: Supplementary file 1 [file TID-21-75-s1.pdf]

SUPPLEMENTARY TABLES:

Supplementary Table 1: Inclusion Criteria for the Parent Oral VAPORS Study Among Young Adults, 2017-2019.

Inclusion Criteria	Exclusion Criteria
Age 18-34years	Not between the ages of 18-34years
Non-users: Do not currently use any tobacco products	Do not own a smartphone
Non-users: Have not used e-cigarette in the past 90 days	Will not remain in the Baltimore area for the next 2 years
Less than 5 pack-years of exposure to tobacco products	Report as HIV positive
Have a smartphone for personal use that is compatible with the VAPORS mobile app	Can not read and speak English
Able to speak and read English	Have a Blood Pressure > 180/100 mmHg
Report as HIV negative	
Not planning to leave the area within the next 2 years	

Supplementary Table 2: Details of Inflammatory Marker Assessment **for the Cross-sectional VAPORS-Endothelial Function Study among Young Adults, 2017-2019.**

Analyte	ELISA Supplier	Intra-assay % coefficients of variability	Inter-assay % coefficients of variability	Limit of detection
Hs CRP	ALPCO	1.54	5.88	0.124 ng/ml
IL-6	RnD Systems	4.77	7.79	0.039 pg/ml
Fibrinogen	AssayPro	1.05	5.20	0.37 μg/ml
P-Selectin	RnD Systems	3.36	9.85	0.5 ng/ml
Myeloperoxidase	RnD Systems	0.37	6.30	1.5 ng/ml

Supplementary Table 3: E-cigarette use Characteristics among E-cigarette Users **in the Cross-sectional VAPORS-Endothelial Function Study among Young Adults, 2017-2019.**

Characteristic	n (%)
Median duration of e-cigarette use (months)	24 (9, 48)
Type e-cigarette/vaping device used	
Rechargeable with a pre-filled cartridge	8 (35)
Device with a refillable tank	8 (35)
Modular system	7 (30)
Frequency of e-cigarette use (times per week)	
Every day	19 (83)

Most days	2 (9)
1-3 days	2 (9)
The flavor of e-cigarettes used most often	
Menthol/mint	9 (39)
Sweet	5 (22)
Fruity	9 (39)
Other	0
The concentration of nicotine used most often	
No Nicotine	1 (4)
Mild nicotine (0.1-0.9%)	8 (35)
Moderate (1-1.9%)	3 (13)
Bold (≥2%)	9 (39)
Don't know/Not sure	2 (9)
How soon after waking use e-cigarettes	
Within 10 minutes	4 (17)
Within 1 hr.	15 (65)
Within 3 hrs.	0
>3 hrs.	4 (17)
Reason for using e-cigarettes	
I like it	11 (48)
To quit smoking or avoid returning to smoking	8 (35)
Believe it's healthier	0
Cheaper	0
Other	4 (17)

© **2023 Boakye E. et al**.
